# Increased Risk of High-Grade Hemorrhage in Cancer Patients Treated with Gemcitabine: A Meta-Analysis of 20 Randomized Controlled Trials

**DOI:** 10.1371/journal.pone.0074872

**Published:** 2013-09-23

**Authors:** Yi Hu, Jingliang Wang, Haitao Tao, Baishou Wu, Jin Sun, Yao Cheng, Weiwei Dong, Ruixin Li

**Affiliations:** Department of Oncology, Chinese PLA General Hospital, BeiJing City, People’s Republic of China; Univeristy of Melbourne, Australia

## Abstract

**Purpose:**

Gemcitabine, a third-generation anticancer agent, has been shown to be active in several solid tumors. High-grade hemorrhage (grade≥3) has been reported with this drug, although the overall risk remains unclear. We conducted a meta-analysis of randomized controlled trials evaluating the incidence and risk of high-grade hemorrhage associated with gemcitabine.

**Methods:**

Pubmed was searched for articles published from January 1, 1990 to December 31, 2012. Eligible studies included prospective randomized controlled phase II and III trials evaluating gemcitabine-based vs non-gemcitabine-based therapy in patients with solid tumors. Data on high-grade hemorrhage were extracted. Overall incidence rates, relative risk (RR), and 95% confidence intervals (CI) were calculated employing fixed- or random-effects models depending on the heterogeneity of included trials.

**Results:**

A total of 6433 patients from 20 trials were included. Among patients treated with gemcitabine-based chemotherapy, the overall incidence of high-grade hemorrhage was 1.7% (95%CI: 0.9–3.1%), and the RR of high-grade hemorrhage was 2.727 (95%CI: 1.581–4.702, p<0.001). Exploratory subgroup analysis revealed the highest RR of hemorrhage in non-small-cell lung cancer (NSCLC) patients (RR: 3.234; 95%CI, 1.678–6.233; *p*<0.001), phase II trials (RR 7.053, 95%CI: 1.591–31.27; *p* = 0.01), trials reported during 2006–2012 (RR: 3.750; 95%CI: 1.735–8.108, *p*<0.001) and gemcitabine used as single agent (RR 7.48; 95%CI: 0.78–71.92, p = 0.081).

**Conclusion:**

Gemcitabine is associated with a significant increase risk of high-grade hemorrhage in patients with solid tumors when compared with non-gemcitabine-based therapy.

## Introduction

High-grade hemorrhage is a significant cause of morbidity and mortality in patients with cancer [1], [2], [3], [4]. Although the presence of malignancy itself and its associated physiologic changes are likely major contributors to an increased risk of hemorrhage, several cancer treatments, including targeted agents, cytotoxic agents, and supportive care medications [5], [6], [7], [8], [9], have also been associated with increased risk of hemorrhage. Since first approved in 1996 for the treatment of unresectable pancreatic carcinoma, gemcitabine, a widely used pyrimidine antimetabolite that interferes with DNA synthesis, has been shown to be active in other solid tumors [10], [11], [12], [13], [14], [15], [16], [17]. Although common adverse events associated with gemcitabine are myelosuppression and mild liver function abnormalities [18], high-grade hemorrhage (grade≥3) has been sporadically reported in several randomized controlled trials (RCTs) [19], [20], [21], [22], [23], [24], [25]. However, the risk of high-grade bleeding in cancer patients receiving gemcitabine that has been reported in clinical trials has not been completely consistent, and none of these trials is large enough to define the overall risk. In addition, an individual trial may be limited to the study of one tumor type. Therefore, we propose that pooling analyses of the current studies may provide a better understanding of the overall risk of high-grade bleeding among cancer patients who receive gemcitabine. As a result, we performed a systematic review and meta-analysis of RCTs to evaluate the incidence and relative risk (RR) of high-grade hemorrhage in cancer patients receiving gemcitabine-based versus non-gemcitabine-based chemotherapy.

## Methods

### Data Source

The selection and systematic review of trials was performed in accordance with the Preferred Reporting Items for Systematic Reviews and Meta-Analysis (PRISMA) statement (see Checklist S1) [26]. Trials were selected from those published in PubMed between January 1, 1990, and December 31, 2012, with “gemcitabine,” “cancer,” “carcinoma”, and “randomized clinical trial” as keywords. Only trials published in peer-reviewed publications in full manuscript form in English were eligible. Only the most recent publication was included when duplicates were identified.

### Study Selection

Our primary objective was to evaluate the association between treatment with gemcitabine-based therapy and high-grade hemorrhage in patients with cancer. Clinical trials meeting the following criteria were included in the meta-analysis: 1) prospective randomized controlled phase II or III trial of cancer patients, 2) random assignment of participants to treatment with gemcitabine or non-gemcitabine-containing therapy, and 3) available data on high-grade hemorrhage. The quality of reports of clinical trials was assessed and calculated using the 5-item Jadad scale including randomization, double-blinding, and withdrawals as previously described [27].

### Data Extraction and Clinical End Point

Data extraction was conducted independently by two investigators (Y.H. and W.J.), and any discrepancy between the reviewers was resolved by consensus. For each study, the following information was extracted: author, publication year, trial phase, treatment arms, number of patients enrolled, number evaluable for toxicity, underlying malignancy, median age, median treatment duration, median progression-free survival, adverse outcomes of interest (high-grade hemorrhagic events), gemcitabine dosage (mg/m^2^). The following adverse outcomes were considered as hemorrhagic events and included in the main analysis: ecchymosis or petechiae; epistaxis; eye hemorrhage; gastrointestinal hemorrhage; gum hemorrhage; injection-site hemorrhage; hematemesis; hematuria; hemoptysis; non-specific hemorrhage; hemothorax; melaena; menorrhagia; metrorrhagia; purpura; rectal hemorrhage; retroperitoneal hemorrhage; CNS hemorrhage; and vaginal hemorrhage (includes menorrhagia and metrorrhagia). We also included (when available) the incidences of high-grade (grade 3 or above) hemorrhagic events. We assessed and recorded adverse events according to the National Cancer Institute’s common toxicity criteria (version 2 or 3), which have been adopted widely in cancer clinical trials [28].

### Statistical Analysis

All analyses were performed using Stata version 12.0 software (Stata Corporation, College Station, Texas, USA) and Open Meta-Analyst software version 4.16.12 (Tufts University, URL http://tuftscaes.org/open_meta/). For the calculation of incidence, the number of patients with high-grade hemorrhagic events and the number of patients receiving gemcitabine were extracted from the selected clinical trials; the proportion of patients with high-grade hemorrhagic events and 95% confidence interval (CI) were derived for each study. For the calculation of relative risk (RR), patients assigned to gemcitabine-based therapy were compared only with those assigned to control treatment in the same trial. Between-study heterogeneity was estimated using the χ^2^-based Q statistic [29]. Heterogeneity was considered statistically significant when *P*_heterogeneity_ <0.1. If heterogeneity existed, data was analyzed using a random effects model (DerSimonian Larid method). In the absence of heterogeneity, a fixed effects model was used (Mantel-Haenszel method). Continuity corrections with 0.5 were adopted for trials with zero events in either or both arms. A two-sided *p*-value less than 0.05 was considered significant. Prespecified subgroup analyses were performed according to tumor type, phase of trials, publication year or treatment regimens. To assess the stability of results, sensitivity analysis was performed by sequential omission of individual studies. The presence of publication bias was evaluated by using the Begg and Egger tests [30], [31].

## Results

### Systematic Literature Search

**Figure 1:**
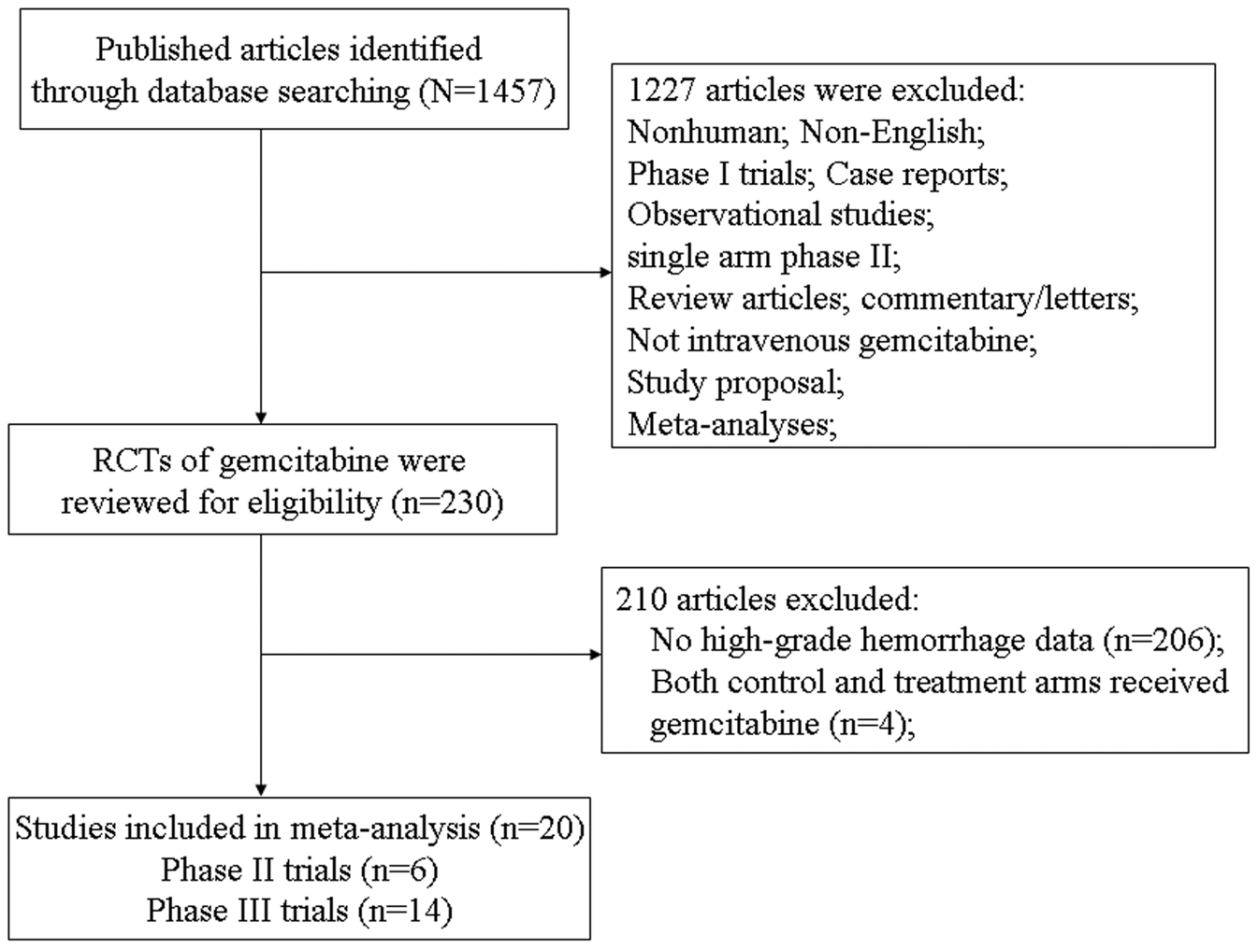
Selection process for randomized controlled trials included in the meta-analysis.

The literature search yielded 1457 publications describing the use of gemcitabine, and 20 RCTs were finally included in the meta-analysis. The selection process is summarized in Figure 1. In total, 6,433 patients were investigated in these trials and they had a variety of cancers: NSCLC (twelve trials) [19], [20], [23], [32], [33], [34], [35], [36], [37], [38], [39], [40], breast cancer (three trials) [24], [41], [42], pancreatic cancer (three trials) [13], [43], [44], bladder cancer (one trial) [21], Carcinoma of unknown (one trial) [45]. All included trials involved randomized treatment allocation. None were placebo controlled or double blind, and the median Jadad score was 2 (range = 2–3). Sample size were in the range of 50 to 1135 patients, with seven trials including >400 patients each. According to the inclusion criteria of each trial, patients were required to have an adequate renal, hepatic and hematologic function. The median age of study participants was in the range of 53–77 years (some studies only reported the mean age). Table 1 reports the study and patient characteristics for the included trials.

**Table 1:** Characteristics of 20 included trials in the meta-analysis (n = 6433).

Studies	Phase	Underlying malignancies	Enrolled patients (n)	Treatment arms	Patients for analysis	Median age (years)	Median treatment duration (months/cycles)	Median PFS/TTP (months)	Median OS (months)	Jadad score	Reported bleeding events
**Cardenal F. et al 1999**	III	NSCLC	135	GEM 1250 mg/m^2^+CDDP q.3.w.	69	59	4.1	6.9	8.7	2	Hemorrhage
				VP-16+CDDP q.3.w.	66	58	3.1	4.3	7.2		
**Crino L. et al 1999**	III	NSCLC	370	GEM 1000 mg/m^2^+CDDP q.4.w.	152	62	4 cycles	5.0	8.6	2	Hemorrhage
				MIC q.4.w.	148	60	4 cycles	4.8	9.6		
**Sandler A.B. et al 2000**	III	NSCLC	522	GEM 1000 mg/m^2^+CDDP q.4.w.	260	62	4 cycles	5.6	9.1	2	Hemorrhage
				CDDP q.4.w.	262	63	2 cycles	3.7	7.6		
**Von der Maase H. et al 2000**	III	Bladder cancer	405	GEM 1000 mg/m^2^+CDDP q.4.w.	203	63	NR	5.8	13.8	2	Hemorrhage
				MVAC q.4.w.	202	63	NR	4.6	14.8		
**Sculier J.P. 2002**	III	NSCLC	284	GEM1000 mg/m^2^+CDDP+CBP q.4.w.	92	NR	86 days	NR	34 weeks	2	Bleeding
				GEM 1000 mg/m^2^+IFO q.4.w.	94	NR	84 days	NR	30 weeks		
				CDDP+CBP+IFO q.4.w.	94	NR	84 days	NR	24 weeks		
**Comella P et al. 2004**	II	NSCLC	264	GEM 1200 mg/m^2^ q.4.w.	68	75	NR	3.3	5.1	3	Bleeding
				PTX q.4.w.	63	72	NR	3.7	6.4		
				GEM 1000 mg/m^2^+PTX q.3.w.	68	72	NR	4.1	9.2		
				GEM+NVB q.3.w.	65	73	NR	4.5	9.7		
**Feher O. et al 2005**	III	MBC	410	GEM 1200 mg/m^2^ q.4.w.	198	69	3.5 cycles	3.4	11.8	2	Hemorrhage
				EPI q.4.w.	199	68	4.6 cycles	6.1	19.1		
**Georgoulias V. et al 2005**	III	NSCLC	413	GEM 1000 mg/m^2^+DOC q.3.w.	197	63	4 cycles	4	9.0	2	GI bleeding
				NVB+CDDP q.3.w.	192	64	4 cycles	5	9.7		
**Zielinski C. et al 2005**	III	MBC	259	GEM 1000 mg/m^2^+EPI+PTX q.3.w.	130	53	7 cycles	9.1	29.5	2	Bleeding
				FU+EPI+CTX q.3.w.	122	54	8 cycles	9.0	24.9		
**Thomas P. et al 2006**	II	NSCLC	100	GEM 1250 mg/m^2^+CBP q.3.w.	51	60	4 cycles	140 days	334 days	2	Bleeding
				NVB+CDDP q.3.w.	49	56	3 cycles	148 days	304 days		
**Oettle H. et al 2007**	III	Pancreatic cancer	368	GEM 1000 mg/m^2^ q.4.w.	186	62	6 cycles	13.4	22.1	3	Bleeding
				Observation	182	61	–	6.9	20.2		
**Ohe Y. et al 2007**	III	NSCLC	602	GEM 1000 mg/m^2^+DDP q.3.w.	151	61	NR	3.2	14.0	2	Cerebral hemorrhage
				PTX+CBP q.3.w.	148	63	NR	3.2	12.3		
				CPT-11+CDDP q.4.w.	147	62	NR	3.3	13.9		
				NVB+DDP q.3.w.	146	61	NR	3.0	11.4		
**Gronberg B.H. et al 2009**	III	NSCLC	436	GEM 1000 mg/m^2^+CBP q.3.w.	217	66	3.1 cycles	NR	7.0	2	Bleeding
				PEM+CBP q.3.w.	219	64	3.3 cycles	NR	7.3		
**Ueno H. et al. 2009**	III	Pancreatic cancer	119	Gemcitabine 1000 mg/m^2^ q.4.w.	57	65	3 cycles	11.4	22.3	2	GI bleeding
				Observation	60	64	–	5.0	18.4		
**Hainsworth J.D. et al. 2010**	III	Carcinoma of unknown primary site	198	GEM 1000 mg/m^2^+CPT-11 q.3.w.	105	59	4 cycles	5.3	8.5	3	Bleeding
				PTX+CBP+ VP-16 q.3.w.	93	61	3 cycles	3.3	7.4		
**Treat J.A. et al 2010**	III	NSCLC	1135	GEM 1000 mg/m^2^+CBP q.3.w.	356	64.1	4 cycles	4.3	7.9	2	Hemorrhage
				GEM 1000 mg/m^2^+PTX q.3.w.	355	64.3	4 cycles	4.5	8.5		
				PTX+CBP q.3.w.	366	64.1	4 cycles	4.7	8.7		
**Brufsky A. et al 2011**	II	MBC	191	GEM 1500 mg/m^2^+PTX+BEV q.4.w.	93	55.2	6 cycles	11.3	24.3	3	Epistaxis, hemorrhage
				PTX+BEV q.4.w.	94	57.5	6 cycles	8.8	25.0		
**Gridlli C. et al 2011**	II	NSCLC	60	GEM 1200 mg/m^2^+SOR q.3.w.	31	74	NR	8.1 weeks	6.6	2	Pulmonary hemorrhage, bleeding
				SOR+ erlotinib	29	76	NR	12.7weeks	12.6		
**EI-Khoueiry A.B. et al 2012**	II	Pancreatic cancer	52	GEM 1000 mg/m^2^+SOR q.3.w.	37	65	2 cycles	2.9	6.5	2	GI bleeding
				SOR	15	66	2 cycles	2.3	4.3		
**Spigel D.R. et al 2012**	II	NSCLC	110	GEM 1500 mg/m^2^+PEM+BEV q.4.w.	55	76	2.5 cycles	4.7	7.5	2	Pulmonary hemorrhage
				PEM+CBP+BEV q.3.w.	55	77	6 cycles	10.2	14.8		

Abbreviation: GEM, gemcitabine; CDDP, cisplatin; VP-16, etoposide; DOC, docetaxel; MIC, Mitomycin+IFO+cisplatin; MVAC, methotrexate+vinblastine+doxorubicin+cisplatin; CBP, carboplatin; IFO, ifosfamide; EPI, epirubicin; NVB, vinorelbine; PTX, paclitaxel; FU, fluorouracil; CTX, cyclophosphamide; PEM, pemetrexed; CPT-11, irinotecan; BEV, bevacizumab; SOR, sorafenib; GI, gastrointestinal tract; NSCLC, non-small-cell lung cancer; MBC; metastatic breast cancer; PFS, progression-free survival; TTP, time-to progression; OS, overall survival; NR, not reported;

### Publication Bias

No evidence of publication bias was detected for the RR of high-grade hemorrhagic events in this study by either Begg or Egger’s test (Begg’s test *p* = 0.81; Egger’s test *p* = 0.21).

### Incidence of High-grade Hemorrhage

**Figure 2:**
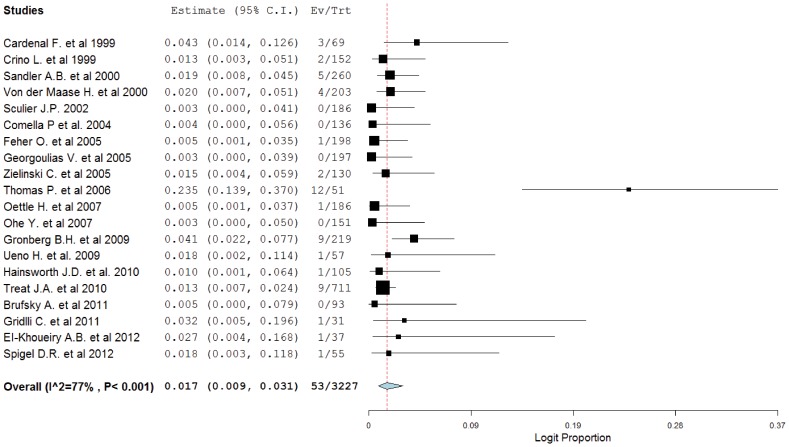
Incidence of high-grade hemorrhagic events associated with gemcitabine.

A total of 6433 patients were included in the analysis. In the gemcitabine group, 53 patients experienced high-grade hemorrhage compared with 18 patients in the non-gemcitabine group. The highest incidence (23.5%; 95% CI, 13.9%–37.0%) as observed in a phase II NSCLC trial [35], and the lowest incidence was observed in five trials in which no hemorrhagic events occurred [23], [33], [34], [36], [42]. Using a random-effects model (heterogeneity test: Q = 81.314; *P*<0.001; *I* ^2^ = 77%), the summary incidence of high-grade hemorrhagic events in patients receiving gemcitabine-based therapy was 1.7% (95% CI, 0.9%–3.1%, Figure 2).

### Relative Risk of High-grade Hemorrhage

**Figure 3:**
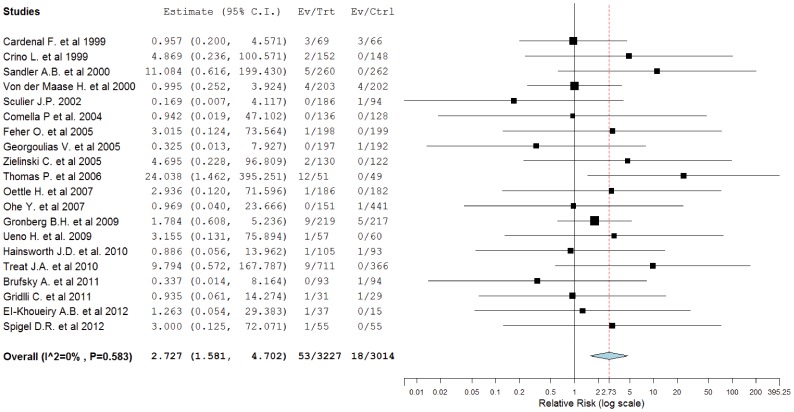
Relative risk of high-grade hemorrhagic events associated with gemcitabine-based vs non-gemcitabine-based therapy.

**Figure 4:**
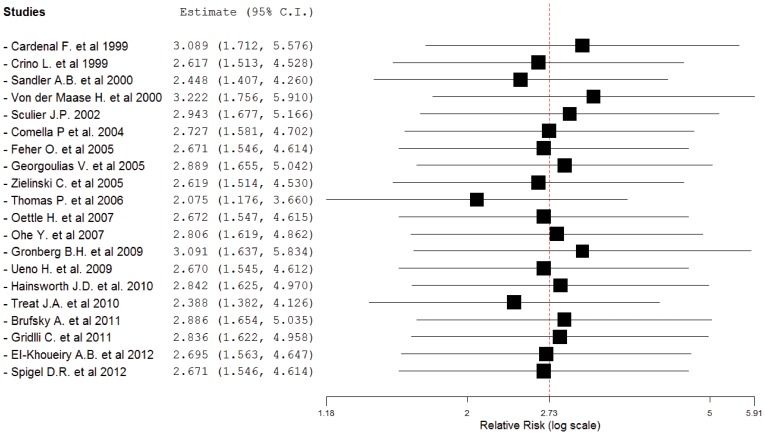
Meta-analysis of high-grade hemorrhagic events associated with gemcitabine-based vs non-gemcitabine-based therapy: “leave-one-out” sensitivity analysis.

To investigate the specific contribution of gemcitabine to the development of hemorrhagic events and exclude the influence of confounding factors such as underlying malignancy, and other therapeutic interventions, we therefore determined the relative risk (RR) of gemcitabine associated hemorrhagic events. The combined results demonstrated that the use of gemcitabine was associated with a significantly increased risk of developing high-grade hemorrhage with a RR of 2.727 (95%CI: 1.581–4.702, p<0.001, Figure 3). We also did sensitivity analysis to examine the stability and reliability of pooled RRs by sequential omission of individual studies. The results indicated that the significance estimate of pooled RRs was not significantly influenced by omitting any single study (Figure 4).

### Influence of Underlying Tumor Type on RR of High-grade Hemorrhage

To better determine possible relationships between gemcitabine and high-grade hemorrhage, we performed several prespecified subgroup analyses, stratifying patients by malignancy, phase of trial and year of publication (Table 2). The incidence of severe hemorrhage was highest among patients with NSCLC (2.0%, 95%CI: 0.8–4.6%), followed by pancreatic cancer (1.4%, 95%CI: 0.4–4.1%) and MBC (1.0%, 95%CI: 0.3–2.7%). The effect sizes varied, and the highest RR of hemorrhage was observed in non-small-cell lung cancer patients (RR: 3.234; 95%CI, 1.678–6.233; *p*<0.001), but the differences among tumor types were not statistically significant.

**Table 2:** Relative risk of high-grade hemorrhage based on prespecified subgroups.

Group	No. of trials	Gemcitabine arm			Non-gemcitabine arm			I^2^,%	Relative risk (95%CI)	*P* for relative risk	*P* for group difference
		No. of events	No. of patients	Incidence (%)	No. of events	No. of patients	Incidence (%)				
**Overall**	20	53	3227	1.7	18	3014	1.2	0%	2.272 (1.581–4.702)	<0.001	NA
**Tumor type**											
NSCLC	12	12	2218	2.0	12	2047	1.0	20%	3.234 (1.678–6.233)	<0.001	0.444
Pancreas	3	3	280	1.4	0	257	0.0	0%	2.259 (0.362–14.12)	0.383	
MBC	3	3	421	1.0	1	415	0.6	0%	2.955 (0.299–29.24)	0.354	
Others	2	5	308	1.7	5	295	1.8	0%	0.972 (0.285–3.321)	0.964	
**Phase of trials**											
Phase II	6	15	403	2.6	2	370	1.4	43%	7.053 (1.591–31.27)	0.01	0.524
Phase III	14	38	2824	1.6	16	2644	0.9	0%	2.211 (1.211–4.038)	0.01	
**Publication year**											
1999–2005	9	17	1531	1.6	9	1413	1.0	0%	1.808 (0.806–4.057)	0.151	0.278
2006–2012	11	36	1696	2.1	9	1601	1.2	0%	3.750 (1.735–8.108)	<0.001	
**Gemcitabine-based regimens** 1											
Single agent	4	3	509	0.8	0	504	0	0%	7.48 (0.78–71.92)	0.081	0.876
Doublet combination	14	47	2413	2.0	17	2237	1.3	8%	2.41 (1.45–3.99)	<0.001	
Triplet combination	4	3	370	1.2	2	365	0.9	0%	1.47 (0.25–8.47)	0.67	

Abbreviation: NSCLC, non-small-cell lung cancer; MBC, metastatic breast cancer;

1 gemcitabine is used as single agent and combination therapy in two clinical trials, thus there is a total of 22 comparisons.

### Influence of Phase of Trials on RR of High-grade Hemorrhage

Given the potentially differing risks of hemorrhage between phase II and III trials, an exploratory analysis stratifying patients by phase of trial was performed (Table 2). Interestingly, the effect size was greater in the phase II trials (RR 7.053, 95%CI: 1.591–31.27) versus phase III trials (RR 2.211, 95%CI: 1.211–4.038). However, there was no significant difference between these subgroups.

### Influence of Publication Year on RR of High-grade Hemorrhage

We hypothesized that the incidence of severe hemorrhage reported in cancer clinical trials may have increased over the past decade. Therefore, we explored the impact of publication year on incidence and RR of severe hemorrhage with gemcitabine-based therapy. Notably, the incidence of hemorrhage in the 9 trials published from 1999 to 2005 was 2.1% (95%CI: 0.8–5.4%), compared with an incidence of 1.6% (95%CI: 0.9–2.6%) in the 11 trials published from 2006 to 2012. In the 11 trials published from 2005 to 2012, gemcitabine-based therapy was associated with an RR of hemorrhage of 3.75 (95%CI, 1.735–8.108). In trials published from 1999 to 2005, gemcitabine-based therapy was associated with an RR of hemorrhage of 1.808 (95%CI, 0.806–4.057). This difference did not reach statistical significance.

### Influence of Treatment Regimes on RR of High-grade Hemorrhage

Concomitant agents with gemcitabine, including bevacizumab and sorafenib, might increase the risk of gemcitabine-related hemorrhage events. We therefore performed sub-group analysis according to gemcitabine-based regimens. An increased risk of hemorrhage events was observed in gemcitabine used as single agent (RR 7.48, 95%∶0.78–71.92), doublet combination (RR 2.41, 95%CI: 1.45–3.99) and triplet combination (RR 1.47, 95%CI: 0.25–8.47) when compared to controls, though the risk did not significantly increase in gemcitabine therapy used as single agent (p = 0.081) and triplet combination (p = 0.67) (Table 2). One possible explanation for this finding was that there were a limited number of trials to investigate the risk of hemorrhage events in gemcitabine used as single agent and triplet combination, thus the power to investigate the risk was small. Interestingly, the effect size was greater in gemcitabine used as single agent versus gemcitabine combination, which suggested that concomitant agents with gemcitabine had limited effects on the risk of gemcitabine-related hemorrhage events.

## Discussion

To our best knowledge, this is the first meta-analysis to investigate the risk of high-grade hemorrhage associated with gemcitabine. Our analysis of data from randomized controlled trials shows a nearly three-times increased risk of high-grade hemorrhage in cancer patients treated with gemcitabine-based therapy. Additionally, the overall incidence of gemcitabine associated high-grade hemorrhagic events is 1.7% (95% CI, 0.9%–3.1%). Based on these results, we could conclude that while the incidence of high-grade hemorrhage in patients treated with gemcitabine is low, the use of gemcitabine is associated with significantly increased risk of high-grade hemorrhage when compared with non-gemcitabine-based therapy. These results would provide important information for clinicians who use gemcitabine to treat patients with solid cancer.

Many factors such as age, race, sex, mobility, underlying cancer, and concurrent use of anticoagulants or chemotherapy are known to contribute to the development of hemorrhage in cancer patients [46]. Thus, we also explore the risk factors for gemcitabine associated hemorrhagic events. Our exploratory subgroup analyses reveal some interesting hypothesis-generating findings. The effect sizes vary with regard to the RR of hemorrhage in specific tumor types, and the highest RR of serious hemorrhage is observed in non-small-cell lung cancer patients (RR: 3.234; 95%CI, 1.678–6.233; *p*<0.001). However, the interpretation of these findings is hampered by the low number of patients and events in certain subgroups. As a result, more high-quality trials are still needed to investigate the risk of gemcitabine associated hemorrhage in these tumors. We hypothesize that the incidence of serious hemorrhage reported in clinical trials over the last decade may have increased because of an increased awareness that serious hemorrhage may be treatment rather than disease related. Indeed, the incidence of serious hemorrhage is higher in trials published between 2006 and 2012 compared with trials published between 1999 and 2005. Then, we also investigate the differing risks of hemorrhage between phase II and III trials. Interestingly, the effect size is greater in the phase II trials versus phase III trials. However, there is no significant difference between these subgroups. Finally, we perform sub-group analysis to detect the influence of concomitant agents on risk of hemorrhage, and find that there is an increased risk of hemorrhage events in gemcitabine used as single agent, doublet combination and triplet combination when compared to controls, though the risk did not significantly increase in gemcitabine therapy used as single agent and triplet combination.

The pathogenesis of gemcitabine-induced hemorrhage remains unclear, gemcitabine-induced thrombocytopenia may be directly related to its increased risk of hemorrhage, but the risk of hemorrhage depends not only the platelet count, but also on the underlying disease, platelet function and complications such as fever and infection or the presence of coagulation defects [47]. It is unknown whether gemcitabine affects the coagulation cascade or endothelial cell. As a result, studies focusing on this issue are still needed.

Our meta-analysis had several limitations. First, this meta-analysis was not based on individual patient data, and meta-analyses based on published data tended to overestimate treatment effects compared with individual patient data analyses. In addition, it precluded a more comprehensive analysis such as adjusting for baseline factors and other differences that existed between the trials from which the data were pooled. Therefore, the results must be interpreted cautiously, as an individual patient data-based meta-analysis would give more reliable estimation than one based on published data. Secondly, trials reported zero high-grade hemorrhage in one or both arms were also included for analysis. In this setting, using fixed effects models and continuity corrections would bias the results towards null. But we felt that including trials reporting zero high-grade hemorrhage would provide the most conservative estimate. Thirdly, different treatment strategy, duration, and regimens contributed to increase the clinical heterogeneity of the meta-analysis, which made the interpretation of the meta-analysis more problematic, although we performed sub-group analysis and sensitive analysis. Additionally, targeted drugs including bevacizumab [7], [9] and sorafenib [8], could increased the risk of hemorrhage, which is another potential bias for evaluating the risk of hemorrhage by gemcitabine.

In conclusion, although the incidence of high-grade hemorrhage in patients treated with gemcitabine is low, a significantly increased risk of high-grade hemorrhage is detected when compared with non-gemcitabine therapy. Clinicians should be cautious when using gemcitabine-based therapy for treating cancer patients, especially those at high risk.

## Supporting Information

Checklist S1 **PRISMA Checklist.**
(DOC)
